# Structurally assisted super black in colourful peacock spiders

**DOI:** 10.1098/rspb.2019.0589

**Published:** 2019-05-15

**Authors:** Dakota E. McCoy, Victoria E. McCoy, Nikolaj K. Mandsberg, Anna V. Shneidman, Joanna Aizenberg, Richard O. Prum, David Haig

**Affiliations:** 1 Department of Organismic and Evolutionary Biology, Harvard University, 26 Oxford Street, Cambridge, MA 02138, USA; 2 Steinmann-Institut für Geologie, Mineralogie und Paläontologie, Universität Bonn, Nussallee 8, 53115 Bonn, Germany; 3 Department of Health Technology, Technical University of Denmark, 2800 Kongens Lyngby, Denmark; 4 John A. Paulson School of Engineering and Applied Sciences, Harvard University, 9 Oxford Street, Cambridge, MA 02138, USA; 5 Department of Chemistry and Chemical Biology, Harvard University, 12 Oxford Street, Cambridge, MA, USA; 6 Kavli Institute for Bionano Science and Technology, Harvard University, 29 Oxford Street, Cambridge, MA, USA; 7 Department of Ecology and Evolutionary Biology, and Peabody Museum of Natural History, Yale University, New Haven, CT 06511, USA

**Keywords:** microlens arrays, structural colour, peacock spiders, sexual selection

## Abstract

Male peacock spiders (*Maratus*, Salticidae) compete to attract female mates using elaborate, sexually selected displays. They evolved both brilliant colour and velvety black. Here, we use scanning electron microscopy, hyperspectral imaging and finite-difference time-domain optical modelling to investigate the deep black surfaces of peacock spiders. We found that super black regions reflect less than 0.5% of light (for a 30° collection angle) in *Maratus speciosus* (0.44%) and *Maratus karrie* (0.35%) owing to microscale structures. Both species evolved unusually high, tightly packed cuticular bumps (microlens arrays), and *M. karrie* has an additional dense covering of black brush-like scales atop the cuticle. Our optical models show that the radius and height of spider microlenses achieve a balance between (i) decreased surface reflectance and (ii) enhanced melanin absorption (through multiple scattering, diffraction out of the acceptance cone of female eyes and increased path length of light through absorbing melanin pigments). The birds of paradise (Paradiseidae), ecological analogues of peacock spiders, also evolved super black near bright colour patches. Super black locally eliminates white specular highlights, reference points used to calibrate colour perception, making nearby colours appear brighter, even luminous, to vertebrates. We propose that this pre-existing, qualitative sensory experience—‘sensory bias’—is also found in spiders, leading to the convergent evolution of super black for mating displays in jumping spiders.

## 1. Background

Colour plays a number of roles in inter- or intra-specific visual signalling, including camouflage, mimicry, warning coloration and social signalling [1]. Some of the most elaborate colour displays have evolved because of sexual selection by mate choice [2–5], exemplified by the peacock spiders (*Maratus*, Salticidae [6]), which are subject to unusually intense sexual selection [7]. Among males, competition to be preferred by females and secure mating opportunities has produced innovative visual traits at multiple size scales [6,8–11]. Investigating these stimulating visual displays can (i) reveal novel colour-producing mechanisms [10,12], (ii) inform our understanding of animals' visual ecology and sensory experiences [8,13], and (iii) guide the design of human-made devices for colour production and other forms of light manipulation [12].

The highly visual, polygynous jumping spiders (Salticidae) have elaborate displays of bright colours and behaviours [6,14]. Particularly, male jumping spiders of the genus *Maratus*, known as peacock spiders, have splendidly coloured abdomens which they erect and wave side-to-side during mating displays to females [6,8,9]. Structural colours in peacock spiders are produced by plate-like blue scales (modified setae) with a dual thin film structure [10] or rainbow scales with two-dimensional diffraction gratings atop a convex three-dimensional microstructure [12]. Brush-like scales produce cream, yellow or red colours through pigments in combination with structural effects [10,14]. Other brush-like black scales contain melanins [12,15]. There is strong mate choice by female peacock spiders for strikingly bright and bold colour patterns; jumping spiders have acute colour vision [16,17] and colourful male ornaments are the direct targets of female choice [7,18,19]. Furthermore, female peacock spiders are extremely choosy and usually mate only once [6]. Therefore, males are under powerful selective pressure to fulfil female preferences.

**Figure 1.**
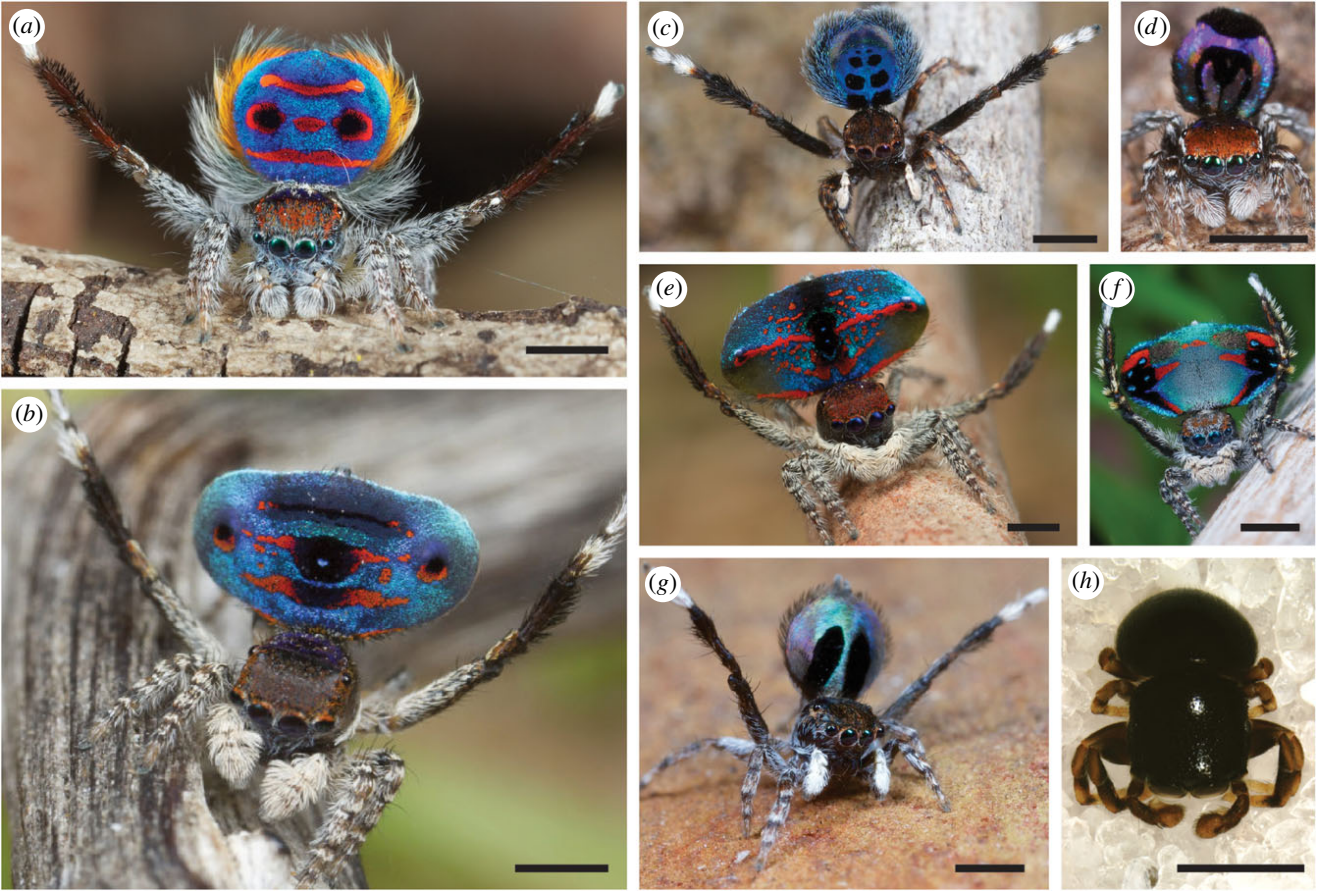
Deep black patches alongside brilliant colours in peacock spiders (*a*–*g*), and a closely related shiny black spider (*h*). (*a*) *Maratus speciosus*, (*b*) *Maratus karrie*, (*c*) *Maratus nigromaculatus*, (*d*) *Maratus robinsoni*, (*e*) *Maratus hortorum*, (*f*) *Maratus avibus*, (*g*) *Maratus chrysomelas* and (*h*) *Cylistella* sp. Scale bars are all 1 mm; for (*a*–*g*), they are estimated based on species-typical size. Scale bars are taken from: (*a*,*b*) specimen measurements herein, (*c*) [20,21], (*d*) [20], (*e*) [22], (*f*) [23], (*g*) [24] and (*h*) Facundo Martín Labarque. Pictures are courtesy of (*a*–*g*) Jürgen Otto and (*h*) Facundo Martín Labarque and may not be reproduced.

Intriguingly, males of many species of peacock spiders have dark, velvety black patches adjacent to bright colour patches (figure 1). This is reminiscent of the super black plumage near bright colours in the birds of paradise (Paradisaeidae), which are also subject to intense sexual selection [25] and have evolved extraordinarily elaborate mating displays [26–30]. Many male birds of paradise evolved deep velvet, ‘super black’ plumages near bright colour [26,28,30,31]; super black is produced by multiple scattering among barbule microstructures which greatly enhances the efficiency of melanin absorption [31]. More generally, super black is defined as structural or structurally assisted absorption with significantly reduced specular reflectance compared to that of a flat (unstructured) surface of the same material [31–33]. In nature, anti-reflection (whether in combination with pigmentary absorption or not) has evolved in moth eyes to reduce glare [34], in transparent aquatic animals to evade detection [35], in glasswing butterflies to avoid avian predators [36], in velvet black spots on a viper to merge into shadows on the forest floor [37] and more—and frequently has inspired anti-reflective engineered materials (e.g. [38]).

Super black coloration is extremely low reflectance (e.g. less than 0.5% directional reflectance in birds of paradise), approaching the darkest human-made materials available [39–41]; this raises the question of why such an intricate, extreme trait evolved. In birds of paradise, super black may have evolved through sensory bias [31], whereby a trait stimulates pre-existing sensory/cognitive biases and preferences in females [4,42,43]. Specifically, in a variety of vertebrates, super black surfaces impede natural mechanisms of colour correction by removing white specular highlights that are used as white-balancing reference points, causing nearby colours to appear brighter—even luminescent [44–46]. Are the velvety black patches in peacock spiders a convergent example of structurally assisted super black for colour emphasis? If so, this implies (i) a widespread sensory bias intrinsic to colour vision in distantly related species, and (ii) a significant role for sensory bias at the extremes of competitive sexual selection.

Here, we characterize the spectral reflectance and surface microstructures of the black areas in two brilliant and boldly patterned species of peacock spiders, *Maratus speciosus* (figure 1*a*) and *Maratus karrie* (figure 1*b*). We use hyperspectral analysis, scanning electron microscopy (SEM) and finite-difference time-domain (FDTD) modelling of the interaction between the structures and incident electromagnetic field. We determine that they use super black, structurally assisted absorption in their displays, which are much less reflective than the normal black cuticle of a closely related normal black spider (*Cylistella* sp., which has no bright colours), and comparable in reflectance to super black bird of paradise plumages. Moreover, we observe a new, distinct type of microstructure in super black spiders different than those previously described in birds of paradise. *Maratus* has brush-like scales similar to the bird of paradise feathers, but also has novel anti-reflective microlens arrays. Based on FDTD modelling, we propose a mechanism for the reduced reflectance and increased light absorption. We further demonstrate that the spiders' microstructural features are roughly at an optimum for the microstructures to achieve minimal reflectance and maximal absorption in the melanin layer.

## 2. Methods

### (a) Specimen details

All spider specimens were obtained from the Harvard Museum of Comparative Zoology Invertebrate Zoology collections, and both bird specimens are from the Yale Peabody Museum of Natural History Ornithological Collections. Note that multiple individual specimens are identified by a single specimen number because they are curated in lots of approximately 3–10 individuals from the same locality and collection date in a single jar.

### (b) Scanning electron microscopy

Spiders were dried, mounted and sputter-coated with 10 nm of Pt/Pd to prepare for SEM. SEM images were taken on an FESEM Ultra55, and measurements were taken from these images using ImageJ. The location of SEM images on the specimens is indicated in the electronic supplementary material, figure S1.

### (c) Hyperspectral imaging

To record reflectance spectra for these spiders, standard spectroscopy could not be used owing to their small size (approx. 2–5 mm in diameter, with even smaller velvety black regions). Therefore, we used a form of microspectrophotometry which captures an image where every pixel encodes a reflectance spectrum between wavelengths 420 and 1000 nm, normalized by a mirror standard (Thorlabs Inc.). We used a Horiba and Cytoviva Model XploRA hyperspectral microscope with MicroManager and ENVI software (issue 4.8). The light source was a DC-950 Fiber-Lite (Colan-Jenner Industries). We used a 50× microscope objective (numerical aperture 0.5) and exposure time of 1000 ms for the super black regions. The mirror standard was too reflective for this exposure time, so we used exposure 100 ms and multiplied all values by 10 (we could perform a linear transformation because the charged-coupled device is a linear detector for the intensities employed). To control for background noise from our instruments, we normalized all measurements by the lamp spectrum; to ensure there was no background noise from ambient conditions, we turned off the light source and took a hyperspectral measurement.

From the resulting hyperspectral images, we averaged 10 reflectance spectra from points that were in focus on the image (limited owing to the curvature of spider bodies). To calculate total %-reflectance, we integrated a loess (locally estimated scatterplot smoothing) curve from wavelengths 420–700 and divided the result by the integral of a perfect mirror reflectance standard with reflectance = 100% for the studied 280 nm wavelength span. We performed this analysis with all three species of spiders and with one species from the bird of paradise (Paradisaeidae), which were previously characterized [31], in order to validate the procedure.

We ensured that the black patches did not reflect in the ultraviolet range through multispectral imaging of one male specimen of each species and a female *M. speciosus* (electronic supplementary material, figure S2).

Specimens stored in ethanol may have changes in colour owing to pigment leaching; before hyperspectral imaging, we allowed the spiders to dry for 60 s in air (surface drying of *Maratus* restores the original colour [20]). Further, we were quantitatively analysing the ‘darkness’ of a region; if melanin had been leached, our measurements of the ‘darkness’ of a region are an underestimation, implying that the super black effect is even more pronounced in live peacock spiders.

### (d) Optical modelling

**Figure 2.**
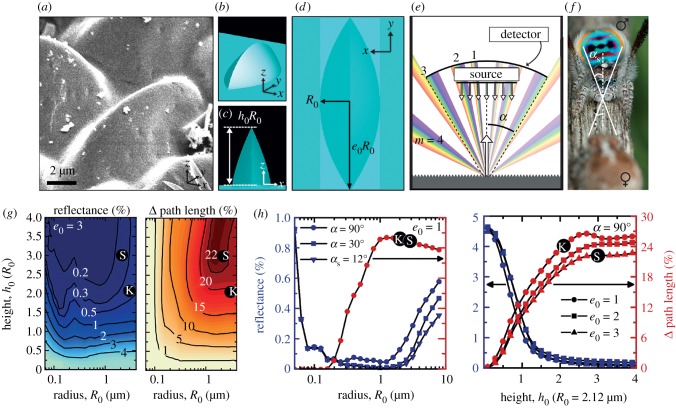
FDTD simulations confirm that spider-like microlens arrays cause path length increase and decrease specular reflectance. (*a*) SEM micrograph of a group of microlenses of *M. speciosus* in a super black region. (*b*) Perspective view of single microlens in the simulation of an infinite hexagonal array. (*c*) *xz* perspective and (*d*) top views of the single microlens, including definitions of the geometrical parameters used in the simulation. (*e*) Schematic of grating-like behaviour (showing orders *m* = 0 through to 4) of the periodic microstructure, with definition of collection angle *α*, where only reflected angles lesser than *α* are collected in the experimental reflectance measurement as well as by the female spider. (*f*) Photograph of male (top)–female (bottom) interaction, with an estimate of the collection angle, *α*, for female spiders, which is determined by considering male abdomen width, female eyes centre-to-centre distance, and courtship distance. The male abdomen is approximately 2.1 mm wide. Photo courtesy of Jürgen Otto and may not be reproduced. (*g*) Contour maps showing the dependence of reflectance (left) and change in path length (right) on the microlens length scales: radius, *R*_0_, and height, *h*_0_, for lens elongation *e*_0_ = 3 and collection angle, *α* = 90°. ‘S’ and ‘K’ approximate the height and radius for *M. speciosus* and *M. karrie*, respectively. (*h*) Reflectance (left axis, blue curves) and change in path length (right axis, red curves) for different *α* (left plot) and *e*_0_ (right plot) and as a function of *R*_0_ (with *e*_0_ = 1, left plot) and *h*_0_ (where *α* = 90°, right plot).

FDTD simulations were performed using the commercially available software Lumerical FDTD, which employs the standard Yee cell method [47] to calculate the spatio-temporal electromagnetic field distribution resulting from an initial pulse launched into the simulation domain. Each real microlens (figure 2*a*) has a superellipsoidal shape (figure 2*b–d*), described by the following function (equation (2.1)), with characteristic structure size, *R*_0_, height, *h*_0_, elongation, *e*_0_, and shape *N* (where *N* = 2 corresponds to an ellipsoid and *N* = 1 is near-pyramidal in the *x*-direction)

2.1 $$z(x,y)=R_{0}h_{0}{\left[1-{\left|\frac{x}{R_{0}}\right|}^{N}-{\left|\frac{y}{R_{0}e_{0}}\right|}^{2}\right]}^{\sqrt[{-2\;\;}]{2N}}.$$

The structures were discretized such that at least 50 mesh elements per half-width were used in each Cartesian direction, with a maximum mesh element size of 30 nm. For the air region outside of the structure, the built-in mesh of 2 was used in the *z*-direction.

In calculating reflectance, three collection angles are of interest: (i) 30° to match the microscope set-up, (ii) 90° to obtain the total reflected light, and (iii) 12°, an estimate of the collection angle of female eyes approximately 0.85 mm from end-to-end facing an approximately 2.1 mm male abdomen sitting approximately 7 mm away (figure 2*e,f*). Although female peacock spider eyes have an impressive field of view of 58° [48], only rays reflected or emitted from the male's abdomen that intersect her eyes are relevant to our work.

For this work, a plane wave was normally incident (*z*-direction) on an infinite array of microstructures in the (*x*,*y*)-plane. The simulation domain was bounded in the *z*-direction by perfectly matched layers (PMLs) while symmetry and antisymmetry boundary conditions were used in the *x* and *y* directions, depending on which polarization was chosen for the incident light. All presented results are averages of two simulations with orthogonal polarization. Frequency domain field monitors were placed above and below the structure to collect the reflected and transmitted light, respectively. A hexagonal packing was chosen in order to emulate the predominant packing observed in the SEMs of the two studied spider species.

The electromagnetic pulse spanned the wavelength range of approximately 350–750 nm (in order to ensure an appreciable field strength in the range of interest, 400–700 nm). PML boundaries and monitors were spaced a distance of at least *λ*_max_/2 apart from each other and from the structure. The simulation was terminated with an auto shutoff level of 10^−4^. The built-in grating projection function was used to decompose the fields collected by the monitors into sets of planar waves travelling in different directions, *θ*. For the reflection, these directions are equivalent to the diffraction angle, where angles larger than the acceptance angle (either defined by the choice of microscopy objective or position of the spiders during courtship) were filtered out. For the transmission, the travelling angles were used to calculate the increase in path length compared to a flat surface, which would not refract normally incident light; the increase in path length is thus given by Δ path length = 1/cos*θ* − 1. The results are presented for wavelengths linearly sampled in steps of 10 nm from the 400–700 nm wavelength span.

The value used in simulations for the refractive index of spider cuticle ranges from 1.5 to 1.63, commonly inferred by identifying a liquid of known refractive index which matches that of the cuticle [9,49,50], thus eliminating structural colours upon immersion. More precise measures of refractive index, for example, Jamin-Lebedoff interference microscopy, find comparable values for butterfly chitin [51], a material related to spider cuticle [48]. We assume that the imaginary component of the refractive index is equivalent to 0, following what was assumed for unpigmented chitin in butterfly wings in [51]. This may contribute to a small overestimation of reflectance, which is preferable to an underestimation because we are here studying the degree to which spider cuticle can be low reflectance. Here, following [10], we use the value of *n* = 1.55 (except where we study the effects of varying *n* in simulation), which is validated by a close match between calculation and measurement (electronic supplementary material, equation S1, see Results).

In peacock spiders, black colour is produced by melanin packaged in spherical pigment granules called melanosomes [15]. In the species studied herein, we identified melanosomes in a dense, disorganized, clumped layer beneath the cuticle (electronic supplementary material, figure S3, ‘Mel’ in figure 4), of the same size and location as melanosomes identified in Hsiung's work on related species [9,15]. For this analysis, we focus on the microstructures but do not specifically model the melanin absorption (see the electronic supplementary material, Methods).

## 3. Results

**Figure 3.**
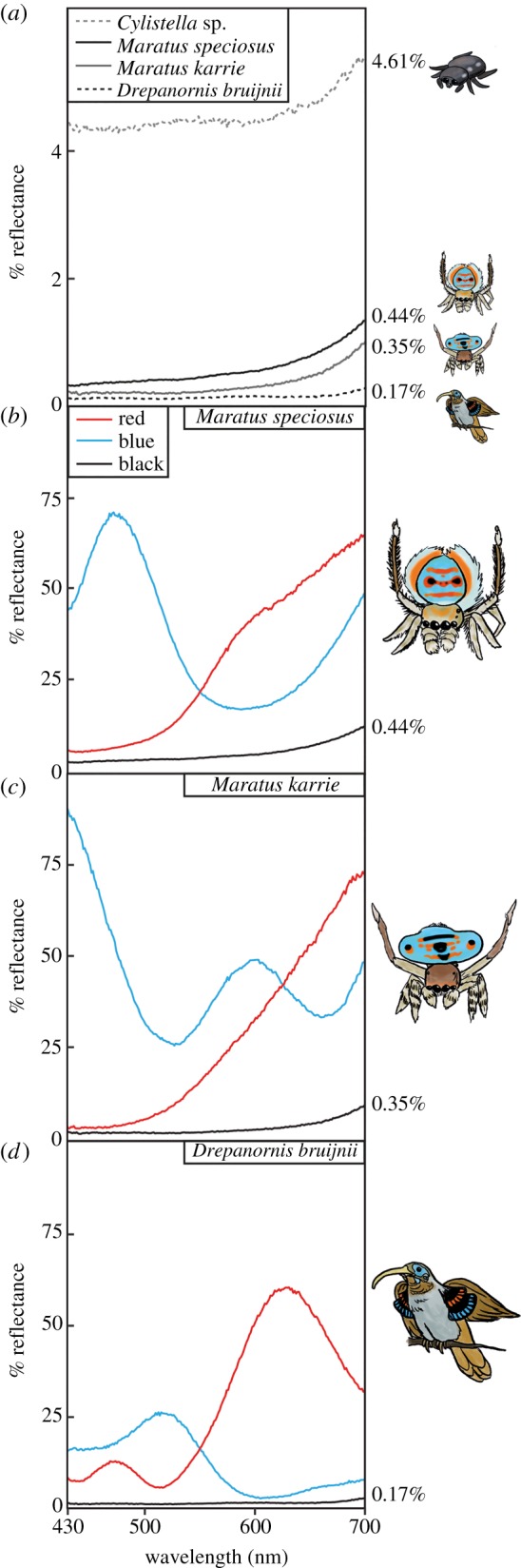
Spectral reflectance measurements using a 30° angle of collection. (*a*) Reflectance curves for typical black spider *Cylistella* sp., super black cuticle in *M. speciosus*, super black cuticle plus super black brush-like scales in *M. karrie* and super black display feathers from bird of paradise *D. bruijnii*. Numbers to the right of the graph represent total per cent reflectance compared to a mirror standard (area under the reflectance curve divided by area under a 100% reflectance curve). (*b*) Reflectance curves for red scales, blue scales and super black regions of *M. speciosus*. (*c*) Reflectance curves for red scales, blue scales and super black regions of *M. karrie*. (*d*) Reflectance curves for red feather tip, blue feather tip and super black feathers of *D. bruijnii.* All measurements were performed with the same hyperspectral imaging set-up, with 50× microscope objective (numerical aperture 0.5). Artwork credit Kay Xia. (Online version in colour.)

Using hyperspectral imaging, we find that the velvety black areas reflect only 0.44% of incident light in *M. speciosus*, and 0.35% in *M. karrie* (figures 1*a*,*b* and 3; electronic supplementary material, figure S1 and table S1, collection angle is 30°), which is similar to values for human-made anti-reflective surfaces [39–41]. These super black patches in *M. speciosus* and *M. karrie* are darker than the normal black cuticle in a closely related, all-black jumping spider (Salticidae) *Cylistella* sp. (4.61% reflectance, figure 3*a*) and brown/black cuticle in *Maratus* (electronic supplementary material, table S1). Super black reflectance in the peacock spiders is comparable to directional reflectance of super black plumage in birds of paradise (figure 3*a*); the bird of paradise measured herein—*Drepanornis bruijnii*, the pale-billed sicklebill—had super black feathers with 0.17% reflectance adjacent to bright red and blue, while other birds of paradise from [31] range from 0.05 to 0.31%.

**Figure 4.**
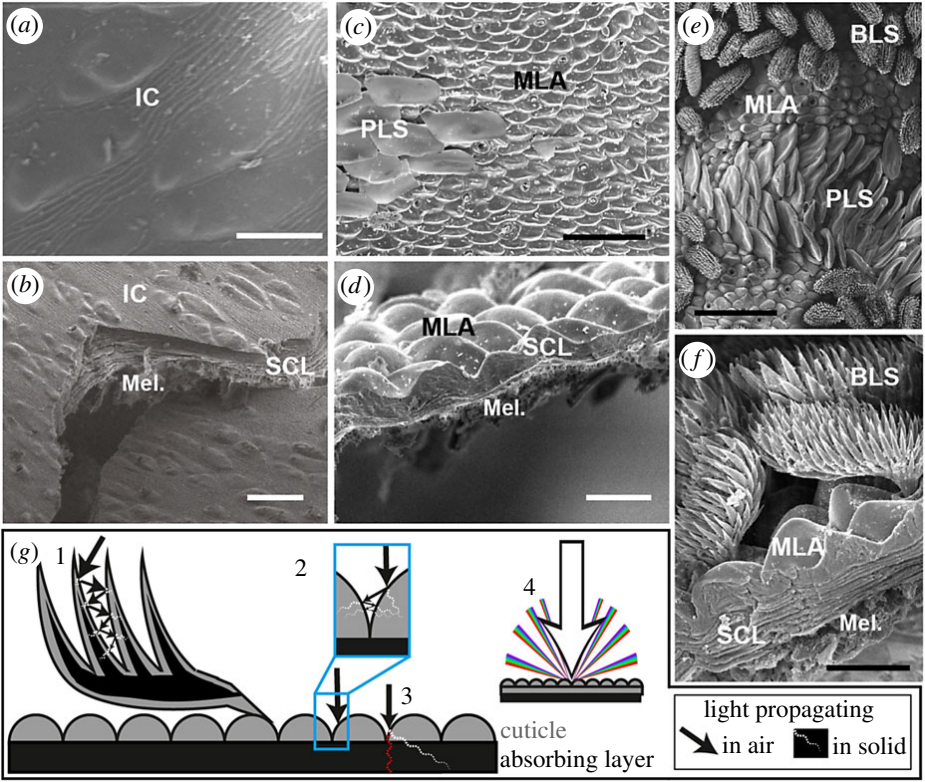
Super black regions in peacock spiders have distinct microstructures compared to normal black regions. (*a*,*b*) SEMs of the brown region of *M. speciosus*, showing the (*a*) surface and (*b*) cross-section. (*c*,*d*) SEMs of super black region in *M. speciosus*, showing the (*c*) surface and (*d*) cross-section*.* (*e,f*) SEMs of super black region in *M. karrie,* showing the (*e*) surface and (*f*) cross-section; BLS, brush-like scales; MLA, microlens array; PLS, blue plate-like scales; IC, irregular cuticle; SLC, striated cuticle layers; Mel., absorbing layer of melanin pigment granules. (*g*) Diagram of the proposed structurally assisted absorption mechanisms by peacock spider microstructures: 1, multiple scattering between spiny projections and iterative absorption as light propagates through cuticle and into the absorbing layer at each scattering event (dotted white line); 2, multiple scattering between bumps and iterative absorption as light propagates through cuticle and into the absorbing layer at each scattering event (dotted white line); 3, increased path length through melanin layers for enhanced absorption (dotted white line) compared with a flat surface (dotted red line); and 4, diffraction of light owing to periodic microlens array, such that less light enters the visual cone of the female spider. Scale bars: (*a*) 30 µm, (*b*) 10 µm, (*c*) 30 µm, (*d*) 10 µm, (*e*) 50 µm and (*f*) 10 µm. The location of SEM images on specimen is indicated in the electronic supplementary material, figure S1.

Using SEM imaging, we identify two types of microstructure present in super black regions of these peacock spiders: cuticular microlens arrays in both and black brush-like scales with many tapering protrusions in *M. karrie* (figure 4). Typical salticid cuticle is smooth and relatively flat and unstructured [48,52] (figure 4*a*,*b*; electronic supplementary material, figure S4), but the cuticle in super black regions of *Maratus* is patterned by microlens arrays with tall, tightly packed, regularly spaced bumps, resembling loose rows of protruding discs or cones (‘MLA’ in figure 4*c*–*f*). The bumps are approximately 6 µm tall in both species, but they are more disc-like in *M. speciosus* and more conical in *M. karrie* (electronic supplementary material, tables S2 and S3)*.* The microlens arrays in super black regions differ from: (i) the irregular and low-relief cuticle in dark brown *Maratus* females, (ii) the flat cuticle in non-display regions of males (figure 4*a*,*b*), and (iii) the smooth unstructured cuticle in the all-black, closely related Salticid spider *Cylistella* (electronic supplementary material, figure S4). In some male *Maratus*, beneath colourful scales, there is relatively flat cuticle patterned with small bumps (electronic supplementary material, figure S5), which ranges in colour from normal black to weak, dark blue [10]. Super black cuticle bumps are significantly taller than this regular bumpy cuticle by 3–4 µm (electronic supplementary material, tables S2 and S3). In human-made materials, taller microlenses are more anti-reflective [53]; therefore, these simple, relatively flat blue or black cuticular bumps may become super black when the bumps increase in height.

Both the microlens arrays and the brush-like scales decrease specular reflectance and enhance melanin-based absorption. The brush-like scales achieve a reflectance of only 0.77% alone (measurement of isolated super black brush-like scale on pale black background; electronic supplementary material, table S1 and figure S5B). We hypothesize that the brush-like scales multiply scatter light between the spiny projections (figure 4*g*, no. 1); at each scattering event, a portion of the light is transmitted into the scale where it is absorbed by melanin pigments, while the remaining portion of the light is reflected at the air–cuticle interface. Rather than being reflected away from the surface of the spider, most of these reflected waves will subsequently encounter another spiny scale projection, where the process is repeated. Thus, multiple scattering causes iterative, near-complete absorption. Super black surface features with many spiny projections have been modelled previously [31], and for two jumping spider genera (*Phidippus* and *Platycryptus*, Salticidae), Hill [54] observed that the shape of dark-pigmented scales ‘minimizes surface glare, thus placing a premium on the interaction of incident light with pigment within the scale’ [54, p. 200]. Therefore, we focused our simulations on the microlenses.

Simulations of light propagation by the surface structures alone accurately model the experimental reflectance for (i) the two peacock spiders (circles labelled S and K on the plots; figure 2*h*) and for (ii) the normal black, unstructured cuticle of *Cylistella* sp. (figure 3; we predicted approx. 4.6% reflectance, consistent with electronic supplementary material, equation S1).

Our numerical simulations confirm that the microlens array surface features decrease specular reflectance (figure 2; electronic supplementary material, figures S6–S8). We describe three optical mechanisms. First, we show that less light is reflected away from the spider's body at the air–cuticle interface; instead, we propose that light is multiply scattered between adjacent lenses, causing iterative absorption (figure 4*g*, no. 2) and a decrease in total surface reflectance. For a flat cuticle surface, reflected light waves scatter back from the surface of the spider causing a brighter appearance. For the cuticular microlens array, reflected light waves frequently encounter another microlens, where some portion of the light is transmitted and absorbed. Through repeated scattering at the air–cuticle interface, less light overall is reflected away from the spider and more light is absorbed as it propagates through the cuticle and absorbing layer (figure 4*g*, dotted white lines). In this manner, the super black regions have less specular reflectance, and less total reflectance, than a comparable flat surface.

Second, our simulations document that the microlens arrays augment light absorption by increasing the path length of light interacting with pigment (figures 2*g,h* and 3*g*, no. 3). The microlens arrays of both *M. karrie* and *M. speciosus* increase the transmitted light path length by 20% compared to an unstructured cuticular surface (figure 2). Such an increase in path length enhances the interaction between the incident light and homogeneous absorbing layer beneath the lens. This would allow the spiders to employ a thinner absorbing layer compared to the thickness required to achieve the same absorption with an unstructured surface. While the melanin granules contribute to scattering as well as absorption, our calculations based on [55] suggest that the relative importance of scattering is low and thus, the path length increase is indeed important for the mechanism of super black (see the electronic supplementary material, Methods).

Third, the microlens arrays reduce specular reflectance by diffracting light out of the viewing cone of a female's eyes (figures 2*e* and 3*g*, no. 4). While the feature size (lens diameter approx. 5–10 µm) is large compared to the wavelength of light, it is still small enough to redirect light into diffraction orders off of normal reflection, thus reducing the portion of light that can enter the acceptance angle of an onlooker's eyes or objective lens (figure 2*e*). This is consistent with observations in measurements of human-made anti-reflective coatings with 2 µm periodicity [53].

Finally, through simulations, we studied how variations in parameters—size, shape, arrangement, refractive index, etc.—could affect the super black phenomenon. Importantly, by sweeping the dimensions of the microlens in simulation, we find that the size and shape of the microlens arrays in the peacock spiders are a balanced optimum between two anti-reflective optical effects: (i) decreased surface reflectance (through diffraction and multiple scattering) and (ii) increased pigmentary absorption (path length increase through the pigmentary layer). Larger microlenses are less efficient at decreasing surface reflectance but more efficient at increasing transmitted light path length (figure 2*g*,*h*). A radius of approximately 2 µm and height of two to three times that radius (approx. 4–6 µm, plotted in figure 2*h* as a function of radius), as observed in these spiders, sits at an optimum trade-off between these two physical effects (figure 2*g*,*h*). Radius and height are most important; variation in refractive index from 1.5 to 1.65 (electronic supplementary material, figure S6), shape *N* from ellipsoid to pyramidal (electronic supplementary material, figure S7) and packing system (the arrangement of microlenses from a top-down view) whether hexagonal versus rectangular (electronic supplementary material, figure S8) had comparably small effects.

To compare the effect of nanostructures versus microstructures, we simulated microlenses with radii ranging from 0.01 to 10 µm. Nanostructures are more effective, i.e. produce lower reflectance, over a wide-angle range (90°), but they do not necessarily perform better when a smaller collection angle is employed, as evident in figure 2*g*,*h*.

## 4. Discussion

Peacock spiders have structurally enhanced, anti-reflective, super black coloration. Brilliantly coloured peacock spiders *M. speciosus* and *M. karrie* produce super black colour owing to microlens arrays on the cuticle (and in *M. karrie*, an overlaying forest of black brush-like scales) above a dense absorbing layer of pigment.

The microlenses of super black cuticle in peacock spiders bear a striking resemblance to anti-reflective surface ornamentation that enhances absorption and reduces specular reflectance in other organisms—including flower petals [56–59], tropical shade plant leaves [60], light-sensitive brittlestar arms [61] and ommatidea in moth eyes [62]. For example, in flowers, conical cells focus incident light and scatter reflected or re-emitted light [63] to produce a velvety coloured appearance and enhance light absorption by the pigment. Applying flower-inspired structures to solar cells (flower power) significantly increased efficiency [64,65]. Flowers and plants evolved simple structures to efficiently harvest light (i) omnidirectionally and (ii) across the visible spectrum (broadband anti-reflection), so they are useful inspiration for broadband and omnidirectional light harvesting [65]. In flowers, as the ratio between microlens height and diameter increases from 0.1 to 0.4, reflection losses drop precipitously [65]. We observe the same pattern in spider microlenses, for which ensembles of taller microlenses are more anti-reflective (figure 2*h*).

Our models show that microlens arrays in spiders behave similarly to engineered microlenses, which are widespread for anti-reflective applications [53,66,67]. The active layer in solar cells is analogous to the dense absorbing layer of melanin beneath the cuticle in *Maratus* spiders (figure 4; electronic supplementary material, figure S3, [15]). Engineers added a microlens array to the light-facing side of a solar cell in order to increase the light absorption efficiency compared to the flat surface by up to 10%: the microlens array reduces optical losses through diffraction and light focusing to increase the path length of light in the active layer [53]. The microlenses in peacock spiders are differently shaped than these engineered microlenses, so it would be informative to simulate optical losses for a solar cell with a spider-inspired ellipsoidal microlens array.

Archetypal anti-reflective surfaces typically have nanostructured features (e.g. moth eyes [34], the glasswing butterfly [36] and black silicon for solar cells [39]), but super black features in peacock spiders and birds of paradise primarily have microstructures. Through our simulations, we investigated the relative performance of microlens arrays ranging in radius from 0.01 to 10 µm. Nanostructures clearly provide a lower reflectance over a wide collection angle (90°), but they lose their advantage at smaller collection angles (figure 2*g*,*h*). During their mating displays, spiders and birds have control over the angle at which they are seen by their potential mate by repositioning their body [11,13,29,31,68]. Thus, males can restrict the collection angle relevant to female eyes; they must be super black only over the viewing cone of a female (estimated herein at 12°; see Methods). On the other hand, in the case of a moth eye, the key evolutionary driving pressure is collecting as much light as possible from all directions to see in low light conditions (as well as to reduce glare in all directions to hide from predators); this gave rise to nanostructures which provide low angle anti-reflection in all directions.

In most organisms, melanin pigments produce normal black colour with white, specular highlights (e.g. glossy hair). By contrast, structural super black in peacock spiders—as well as birds [31], butterflies [69], snakes [37] and human-made materials [32]—creates a featureless black surface with no highlights. Generally, super black seems always adjacent to bright colour in peacock spiders (herein, adjacent to red and blue: figures 1 and 3*b*–*d*) and birds of paradise [31]. The convergent evolution of structurally absorbing black coloration for colourful sexual display by both birds of paradise and now peacock spiders suggests that broadband, featureless black surfaces play an important sensory role in colourful displays for distantly related, but ecologically similar, species.

We hypothesize that super black evolved in peacock spiders and birds of paradise convergently through a shared sensory bias intrinsic to colour perception. According to sensory bias theory, an adaptive feature of the sensory or cognitive system may give rise to a novel or inherently stimulating perceptual experience in the context of social or sexual signalling [70]. Here, we suggest that colour vision in spiders, as in vertebrates, has the adaptive feature for colour correction which gives rise to an intrinsic sensory bias stimulated by super black near brilliant colour. Vertebrates use specular highlights, or gleams from object surfaces, to estimate the magnitude and spectrum of the ambient light illuminating the visual scene, and ‘white balance’ their colour perceptions based on this information [46]. Super black essentially eliminates specular reference points. In vertebrates (specifically humans and goldfish), anti-reflective black surfaces impede the observer's ability to adjust for the amount of ambient light [44,45,71], causing colourful patches to appear self-luminous or popping above the plane of the image. This perceptual illusion is similar to the well-studied Adelson's checker-shadow [72], in which the context around a grey square greatly influences our perception of its brightness. Furthermore, anti-reflective surface features have been shown to enhance the brilliance and saturation of pigmentary colours in snapdragons (*Antirrhinum majus* [57]) and plastic polymers [73]. Super black surrounding or adjacent to bright colour would have the same chromatic effect. Therefore, we hypothesize that the adaptive trait of colour correction also produces an intrinsic sensory/cognitive bias; males in extreme competition for mating may be able to produce impossibly bright colours by stimulating this intrinsic bias through super black.

In both birds and spiders, sexual selection has apparently led to the evolution of a convergent optical, often angle-dependent, illusion—the use of super black structurally assisted absorption to enhance the perceived brilliance of adjacent colours. Super black reveals a fundamental, and broadly distributed, sensory bias.

## Supplementary Material

Electronic Supplementary Material

## Data accessibility

Data available from the Dryad Digital Repository: https://doi.org/10.5061/dryad.m9n940m [74].
