# Supplementary material for: Structurally assisted super black in colourful peacock spiders
Source: Proc Biol Sci. 2019 May 15;286(1902):20190589. doi: 10.1098/rspb.2019.0589 (PMC6532503; doi:10.1098/rspb.2019.0589)
Supplement: Electronic Supplementary Material [file rspb20190589supp1.pdf]

Supplementary Information for

Structurally assisted super black in colorful peacock spiders

Dakota E. McCoy, Victoria E. McCoy, Nikolaj K. Mandsberg, Anna V. Shneidman, Joanna Aizenberg, Richard O. Prum, David A. Haig

Dakota McCoy
Email: dakotamccoy@g.harvard.edu

Contents:

Supplemental Methods
Figs. S1 to S8
Equation S1
Tables S1 to S3

Supplemental Methods

We note that since the absorbing layer of melanin is beneath the simulated structure (see below), it can be treated separately. We use simulations to determine the effect of the microstructuration alone (which does not require quantifying the melanin contribution); this allowed us to save significantly on computation time and therefore perform sweeps over the geometrical parameters. First, we simulated the extent to which microstructures reduce surface reflectance at the air-cuticle interface. To isolate the effect of structure, we assumed that all light transmitted through the cuticle into the spider's body was transmitted or absorbed with no re-emission. This assumption was validated by a close match between simulation and experimentally measured values (see Results and Discussion). Second, inspired by modern solar cells [46,47], we simulated the degree to which microlenses would increase the path length through the melanin-rich, absorbing layer beneath the cuticle. For this calculation, we are not quantifying the chemical absorption of melanin; rather, we are quantifying the path length increase of light through a melanin-rich layer (Fig. S3, [1]) due to the microlens surface features. Given an absorbing medium, path length through that medium strongly impacts total reflectance, so any change in path length would predictably impact appearance.

It is important to note that the melanin herein is packaged in roughly spherical granules, which contribute to scattering (as well as to absorption). To determine whether an increase in path length would be important to the mechanism of super black, we compared three length scales according to the supplemental information of [2]: the melanin layer thickness, the absorption length (average path length over which light attenuates by a factor of e, the base of the natural logarithm), and the scattering length (average distance travelled in the material between successive scattering events). If either the scattering or absorption length is significantly smaller than the thickness of the melanin layer, an increase in path length would not

be important, as the light is either scrambled via scattering or absorbed by the time it reaches the base of the absorbing layer. We find that the absorption and scattering lengths are on the order of 1 µm and 7 µm, respectively, for all wavelengths, while the thickness of the melanin layer is approximately ~2.6 µm (Fig. S3, 3 sphere diameters, each of which is ~870 nm). These numbers suggest that the scattering is not significant over the layer thickness. While the absorption length is smaller than the thickness, it nonetheless leaves an appreciable amount of light available to continue through the melanin layer. This leads us to conclude that the calculated path length increase (see Results) likely contributes to the mechanism of super black production by enhancing the melanin-light interaction (and may allow for lower melanin production than would otherwise be necessary to achieve the same level of absorption).

References

1. Hsiung B-K, Blackledge TA, Shawkey MD. 2015 Spiders do have melanin after all. *J. Exp. Biol.* **218**, 3632–3635.
2. Xiao M, Li Y, Allen MC, Deheyn DD, Yue X, Zhao J, Gianneschi NC, Shawkey MD, Dhinojwala A. 2015 Bio-inspired structural colors produced via self-assembly of synthetic melanin nanoparticles. *ACS Nano* **9**, 5454–5460.

Figure S1

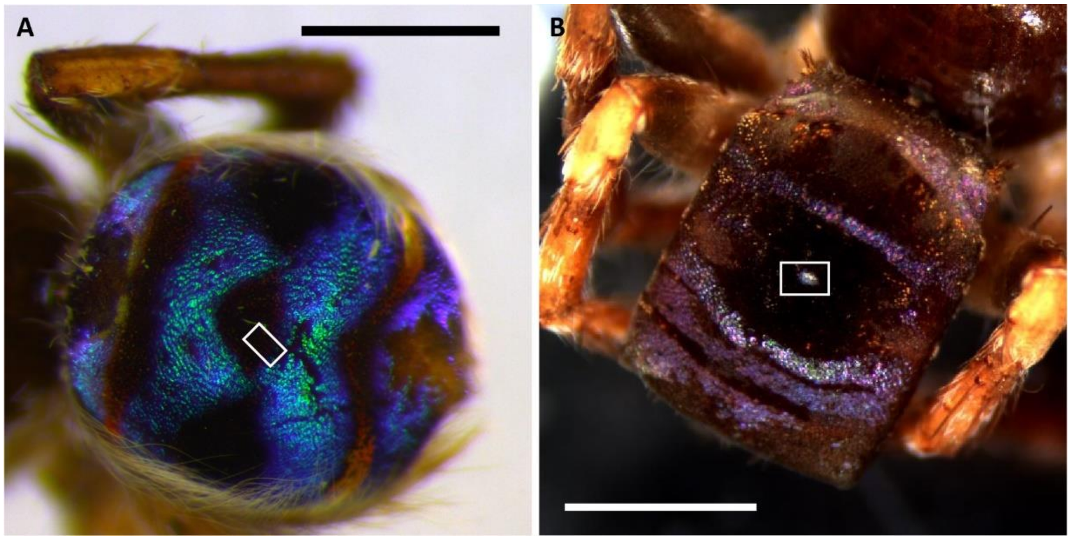

Figure S1: Light microscope images of specimens with approximate location of region shown in SEMs in Fig. 4C-F. (A) *Maratus speciosus,* image credit Mary K. Salcedo. **(B)** *Maratus karrie,* image credit Mara Laslo. Scale bars are 1 mm.

Figure S2

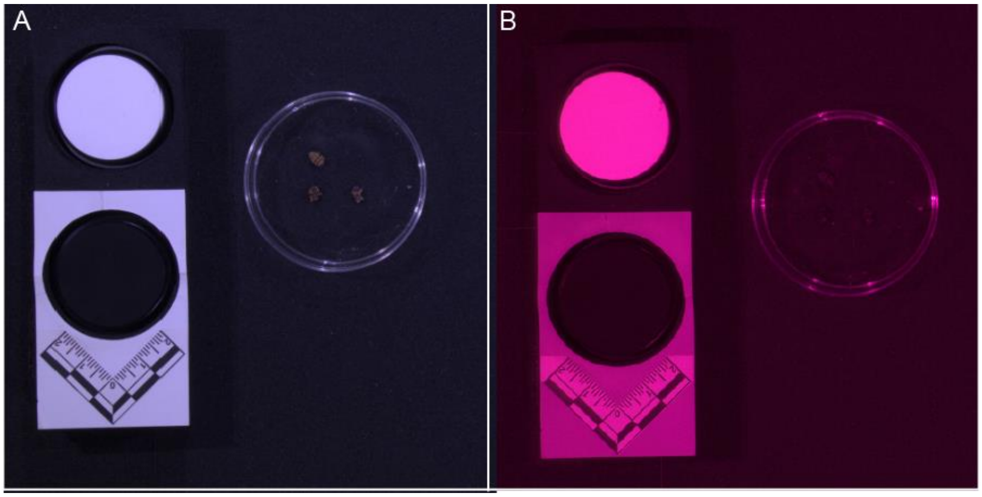

Figure S2. Black regions of the spiders do not reflect UV light. We imaged a female *M. speciosus* (in petri dish, top) a male *M. karrie* (in petri dish, bottom left) and a male *M. speciosus* (in petri dish, bottom right) to test whether the male's black patches reflected UV. **(A)** Spiders illuminated under white light versus **(B)** spiders imaged under UV light. Ruler is measured in cm; total length of one ruler arm = 3 cm.

Figure S3

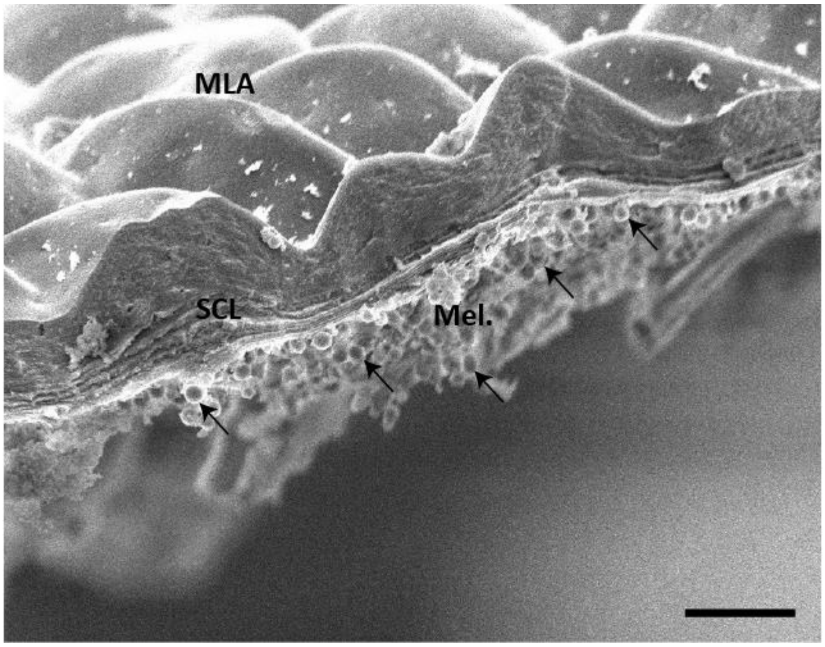

Figure S3: Melanin is packaged in melanosomes, pigment granules, beneath the cuticle. SEM image of *Maratus speciosus* reveals melanosomes. MLA is microlens array, SCL is striated cuticle layers, and Mel. is the melanin-rich absorbing layer. Arrows point to examples of individual pigment granules. Scale bar is 5 µm.

Figure S4

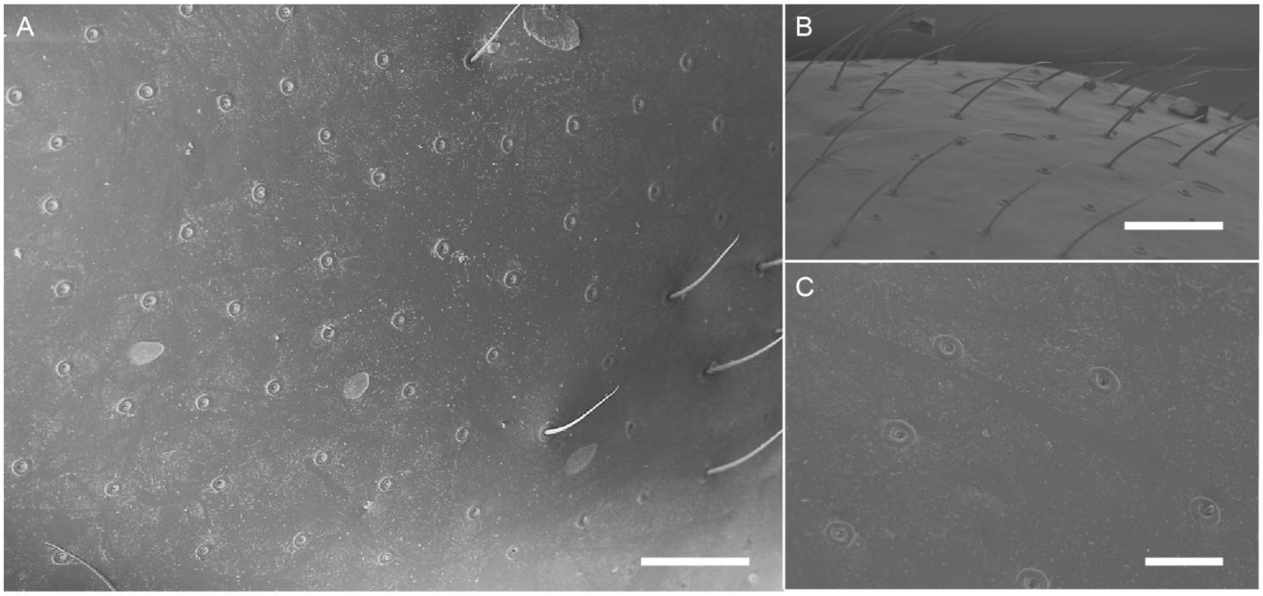

Figure S4. ***Cylistella*** **(a normal black spider, Salticidae) has flat, unstructured cuticle. (A)** Overview of cuticle; simple hair-like scales visible at right. **(B)** Sidelong view of cuticle, showing flat unstructured profile. **(C)** Close-up view of cuticle. Scale bars are (A) 50 µm, (B) 50 µm, and (C) 20 µm.

Figure S5.

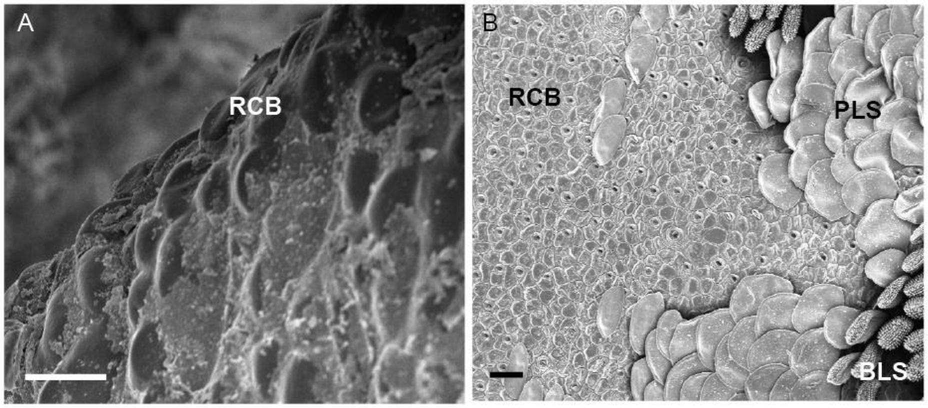

Figure S5: Additional cuticle morphologies generate blue or pale black in peacock spiders. (A) Low-relief bumps produce blue in *Maratus splendens*. **(B)** Low-relief bumps produce pale black in *Maratus karrie.* Regular cuticle bumps (RCB), cuticle microlens arrays (MLA), brush-like scales (BLS), plate-like scales (PLS). Scale bars = 10 µm in (A) and 20 µm in (B).

Figure S6

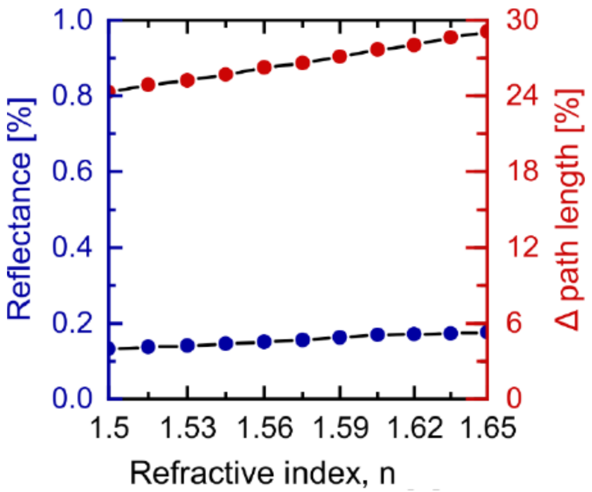

Figure S6: Reflectance and change in path length depend weakly on the cuticle refraction index, $\boldsymbol{n}$. Simulation conducted with radius $R_0 = 2.12$ µm, shape $N = 1$ (which can vary from N = 1, near-pyramidal, to N = 2, ellipsoid), $e_0 = 1$, and height $h_0 = 3$ in a hexagonal array.

Figure S7

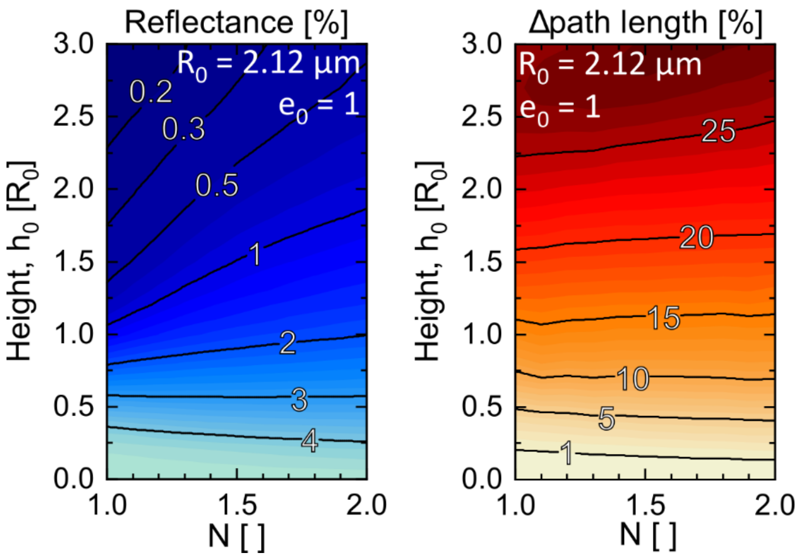

Figure S7: Reflectance and change in path length depend strongly on microlens height and moderately on microlens shape. Shape parameter N = 1 refers to near-pyramidal shape while N = 2 refers to ellipsoids, and h_0 refers to structure height. The simulation was conducted with radius $R_0 = 2.12$ µm and elongation $e_0 = 1$ in a hexagonal array.

Figure S8

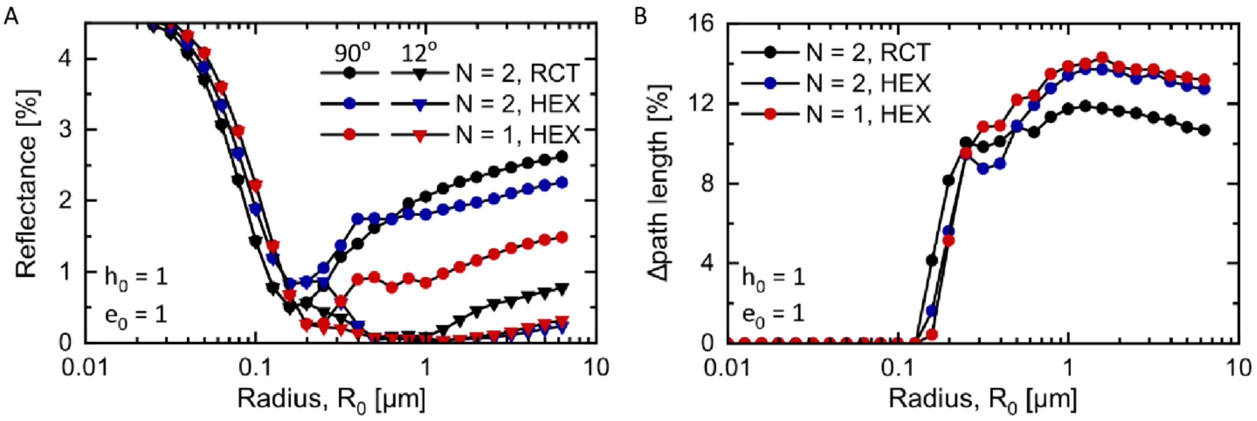

Figure S8: The influence of microlens packing arrangement on reflectance and on change in path length. Change in (A) reflectance and (B) path length by packing arrangement, shape, and radius. Packing arrangement refers to the arrangement of microlenses from a top-down view; packing is either hexagonal (HEX) or rectangular (RCT). Scanning electron micrographs indicate that the microlenses are arranged in an imperfect hexagonal lattice. Shape parameter, N, describes the range from near-pyramidal shape (N = 1) to ellipsoid shape (N = 2). Effects on the reflectance (left) and path length (right) are shown at microstructure sizes where radius R_0 ranges from 10 nm to 6.3 μm. 90° vs 12° refer to the collection angle employed; 90° describes a hemispherical collection angle while 12° approximates the viewing cone of a female spider.

Equation S1

$$R = \left|\frac{n_1 - n_2}{n_1 + n_2}\right|^2 = \left|\frac{1-1.55}{1+1.55}\right|^2 = 0.0465 \qquad \textbf{(Eq. S1)}$$

In this Fresnel equation for normal incidence, R is the reflectance, n_1 is the refractive index of the incidence medium (air in this case, n_1=1) and n_2 is the refractive index of the medium onto which light is incident (cuticle, n_2=1.55; see Fig. S6 for simulation results over a range in refractive index).

Table S1.

Total % reflectance in the visible spectrum for all normal and super black regions measured. Each measurement is the average of 10 points samples from a hyperspectral image, and "n" represents the number of regions measured across all available individuals. SD is standard deviation.

			**% Reflectance**		
**Catalogue Number**	**Species**	**Region**	**mean**	**SD**	**n**
MCZ 101290, MCZ 101292	*Maratus speciosus*	super black cuticle	0.44	0.12	6
MCZ 101295	*Maratus karrie*	isolated super black scale	0.77	0.00092	2
MCZ 101295	*Maratus karrie*	super black scales atop super black cuticle, adjacent to brilliantly reflective spot	0.35		1
MCZ 101295	*Maratus karrie*	lighter black cuticle (not adjacent to brilliantly reflective spot)	1.49		1
MCZ 125016	*Cylistella sp.*	normal black cuticle	4.61	0.32	4
MCZ 110057	*Maratus splendens*	brown/black cuticle	3.10		1
MCZ 101290	*Maratus speciosus* female	brown cuticle	2.61	0.90	2
YPM 75321, YPM 75323	*Drepanornis bruijnii* (Paradisaeidae, Aves)	display feather	0.17	0.01	2

Table S2.
Measurements of structural features (in µm). SD is standard deviation.

	Maratus karrie		*Maratus speciosus*		*Maratus splendens*	
	mean	SD	mean	SD	mean	SD
**Cuticular Microlens Bumps**						
	**super black**					
bump height (cross section) (n=15)	6.07	0.77	6.34	0.73	--	--
super black cuticle: length (n=25)	7.12	1.56	12.99	1.18	--	--
super black cuticle: width (n=25)	5.88	1.54	4.25	0.61	--	--
	**normal black**				**dark blue**	
bump height (cross section) (n=15)	--	--	--	--	2.69	0.62
dark cuticle: length (n=25)	9.31	1.49	9.09	2.26	9.93	1.87
dark cuticle: width (n=25)	7.91	1.32	9.31	1.27	8.97	1.40

Table S3: T-test results for comparisons of structural features.

Parameter tested	Species	Average	p-value	95% CI for difference	n
Super black bump height	*M. speciosus*	6.34±0.73 µm	0.37	[-0.349, 0.89]	15
	M. karrie	6.07±0.77 µm			
**Conclusion**	**The bumps are equally tall in *M. karrie* and *M. speciosus***				
Bump length	*M. speciosus*	12.99±1.18 µm	p<0.0005	[5.09,6.66]	25
	M. karrie	7.12±1.56 µm			
Bump width	*M. speciosus*	4.25±0.61µm	p<0.0005	[-2.30,-0.95]	25
	M. karrie	5.88±1.54 µm			
**Conclusion**	**The bumps are more disc-like (longer and narrower when viewed from above) in *M. speciosus* versus the cone-like bumps of *M. karrie***				
Super black vs. normal black bump height	*M. splendens*	2.69±0.62 µm	p<0.0005	[3.08, 4.21]	25
	M. speciosus	6.34±0.73 µm			
	M. splendens	2.69±0.62 µm	p<0.0005	[2.85, 3.90]	25
	M. karrie	6.07±0.77 µm			
**Conclusion**	**The super black bumps are ~3-4 µm taller than the previously described *M. splendens* blue bumps**				
